# Sulfamethoxazole (SMX) Alters Immune and Apoptotic Endpoints in Developing Zebrafish *(Danio rerio*)

**DOI:** 10.3390/toxics11020178

**Published:** 2023-02-14

**Authors:** Nazish Iftikhar, Isaac Konig, Cole English, Emma Ivantsova, Christopher L. Souders, Imran Hashmi, Christopher J. Martyniuk

**Affiliations:** 1 Institute of Environmental Sciences and Engineering, School of Civil and Environmental Engineering, National University of Sciences and Technology, Sector H-12, Islamabad 44000, Pakistan; 2 Center for Environmental and Human Toxicology, Department of Physiological Sciences, College of Veterinary Medicine, University of Florida, Gainesville, FL 32611, USA; 3 Department of Chemistry, Federal University of Lavras (UFLA), Lavras 37203-202, Minas Gerais, Brazil; 4 UF Genetics Institute and Interdisciplinary Program in Biomedical Sciences Neuroscience, University of Florida, Gainesville, FL 32611, USA

**Keywords:** antibiotics, apoptosis, sulfamethoxazole, immune system, zebrafish

## Abstract

Sulfamethoxazole (SMX) is a broad-range bacteriostatic antibiotic widely used in animal and fish farming and is also employed in human medicine. These antibiotics can ultimately end up in the aquatic ecosystem and affect non-target organisms such as fish. To discern the effect of SMX on developing zebrafish embryos and larvae, we investigated a broad range of sub-lethal toxicity endpoints. Higher concentrations of SMX affected survivability, caused hatch delay, and induced malformations including edema of the yolk sac, pericardial effusion, bent tail, and curved spine in developing embryos. Lower levels of SMX provoked an inflammatory response in larvae at seven days post fertilization (dpf), as noted by up-regulation of interferon (*ifn-γ*) and interleukin 1β (*il-1β*). SMX also increased the expression of genes related to apoptosis, including BCL2-Associated Agonist of Cell Death (*bad*) and BCL2 Associated X, Apoptosis Regulator (*bax*) at 50 µg/L and decreased caspase 3 (*casp3*) expression in a dose-dependent manner. SMX induced hyperactivity in larval fish at 500 and 2500 µg/L based upon the light/dark preference test. Collectively, this study revealed that exposure to SMX can disrupt the immune system by altering host defense mechanisms as well as transcripts related to apoptosis. These data improve understanding of antibiotic chemical toxicity in aquatic organisms and serves as a baseline for in-depth environmental risk assessment of SMX and antibiotics.

## 1. Introduction

The intensity of fish aquaculture and climate change have led to increased disease outbreaks in local production [1]. Bacterial diseases are now recognized as a significant factor resulting in the loss of productivity in the aquaculture industry by causing growth retardation and a higher rate of mortality in fish [2]. As such, bacterial and viral diseases have emerged as a great concern for intensive fish production and aquaculture development. To address this pressing issue, several antibiotics are applied in aquaculture to prevent, control, and treat fish diseases [3].

Antibiotics have received increasing attention in the last decade as an emerging contaminant with the potential to negatively impact human health and the environment. Indeed, significant amounts of antibiotics are currently entering the aquatic environment, resulting in growing environmental concerns [4]. For instance, residual antibiotics dispersed in the environment are highly likely to induce multi-resistant genes in microorganisms and eventually cause adverse ecological and health impacts [5].

First used in 1932 for medical purposes, sulfonamides are one of the concerning antimicrobial classes for the environment. These antibiotics are extensively applied in both human and animal medicine (including aquaculture) because of their broad bactericidal spectrum and cheaper cost relative to other antibiotics [6]. Sulfamethoxazole (SMX), a prevalent member of the class, acts to inhibit the enzymatic pathway involved in bacterial folate production. By regulating the dihydrofolate synthetase enzyme, long-acting SMX prevents para-aminobenzoic acid from being converted into dihydrofolic acid; thus, exerting its bactericidal effect [7].

Sulfonamides, including SMX, trimethoprim, and sulfadimethoxine, are present in surface waters worldwide. Sulfonamides are commonly detected in Asian waste waters, primarily from pig farms [8]. Sulfonamide utilization was reported to be 7890 tons in 2013, with significant concentrations in rivers as most of the wastewater treatment plants are unable to remove various forms of sulfonamides efficiently [9]. SMX is one of the antibiotics that is eliminated in wastewater treatment plants with the least efficiency. Reported concentrations of SMX in hospital effluents are 0.4–2 mg/L in New Mexico, USA [10] and 0.047–309 µg/L in the Republic of Korea [11]. Additionally, SMX was reported in groundwater in the United States of America (0.015–18 µg/L) [12], Pakistan (318 µg/L–16 mg/L) [13] and China (8 ng/L–200 µg/L) [14]. Taken together, SMX can be detected in several water systems on a global scale.

Excessive use of antibiotics, particularly SMX, has been related to several adverse effects in fish, including developmental delays, immunodeficiency, genotoxicity, and histopathological changes [15]. SMX can also impact the physiology of freshwater fish, causing hematological and biochemical disturbances following SMX exposure at environmentally relevant concentrations [16]. Other adverse outcomes include the induction of reactive oxygen species (ROS) in zebrafish (*Danio rerio*) exposed to SMX at 100 µg/L [17]. Moreover, chronic exposure to SMX at 200 μg/L caused a decrease in the body weight of zebrafish, indicating growth-related effects [18].

There is also increasing evidence that the immune system of fish responds to low levels of antibiotic exposure in the environment. In adult Nile Tilapia (*Oreochromis niloticus*), exposure to 0.26 µg/L SMX promoted the expression of inflammatory cytokines [3]. Additionally, immunological toxicity is reported to be more prevalent in the embryo-to-larval period than in the adult phases [19], raising concern about a cascade of toxic events in fish. In this sense, although there is evidence that SMX exposure induces behavioral and histopathological abnormalities in adult fish [20], there are a lack of toxicity data for the early developmental stages of fish species.

There are significant human health issues related to antibiotics in the aquatic environment. For example, the exposure of fish to antibiotics can lead to deposits of sulfonamides in edible animal tissues. Studies have quantified sulfonamides in fish muscles and liver [14,21]. Therefore, residues may culminate in human tissue, leading to bacterial resistance. Moreover, some members of the sulfonamide group of antibiotics are confirmed to be oncogenic [22]. In terms of human safety, the maximum residue limit (100 µg/kg) in edible tissues of sulfonamides has been set by the European Union and the United States [23]. However, a recent study on *Cyprinus carpio*, a freshwater fish, has reported bioaccumulation of SMX at exposure concentrations as high as 25 µg/L. Therefore, continued diligence is warranted regarding the accumulation and toxicity of antibiotics in aquatic organisms.

This study conducted several toxicity assays over a range of SMX concentrations to comprehensively evaluate the effects of SMX on early developmental stages of zebrafish. Zebrafish embryos were exposed to SMX at concentrations ranging from 25 up to 5000 µg/L. Several endpoints related to malformations, survival, hatchability, mitochondrial bioenergetics, apoptosis, reactive oxygen species, gene expression, and behavior (locomotor, light/dark preference) were measured. Based upon the mechanism of action of SMX and due to its immunosuppressive characteristics, we hypothesized that innate immunity and antioxidant pathways would be targeted in zebrafish following exposure to this antibiotic.

## 2. Materials and Methods

### 2.1. Chemical Preparation

Sulfamethoxazole (CAS number: 723-46-6, purity > 99%) was purchased from Sigma Aldrich (St. Louis, MO, USA). A stock solution (400 µg/mL) was prepared in embryo-rearing media (ERM) and stored at −20 °C in amber vials. Test solutions were prepared fresh each day before the setup of all experiments to yield final concentrations of 0, 25, 50, 100, 200, 500, 1000, 2500, and 5000 µg/L of SMX, which were used depending on the endpoint measured (outlined below in each section).

### 2.2. Zebrafish Husbandry

The Cancer-Genetics Research Center (UF) reared adult zebrafish *Danio rerio* (AB × Tübingen). Fish are fed an standardized commercial diet (Zeigler Brothers, Gardners, PA, USA). Zebrafish staging recommendations followed established protocols [24]. Adults were maintained at a temperature of 28 ± 1 °C, a photoperiod of 14L:10D (light–dark), a dissolved oxygen level greater than 6.0 ppm, and at a pH of 7.2 ± 1. The night before embryo collection, two pairs of adult zebrafish (2 males and 2 females), approx. 6 months of age, were transferred to a breeding tank. Male and female zebrafish were separated overnight with a divider. Dividers were removed in the morning to initiate spawning. All experiments were performed at the Aquatic Toxicity Center using the collected eggs. All zebrafish experiments were approved by the Institutional Animal Care and Use Committee (UF IACUC#201708562) of the University of Florida.

### 2.3. Exposure Experiments with Sulfamethoxazole

Embryos (~6 h post-fertilization, hpf) were collected and washed three times using ERM in petri dishes to remove any contamination from breeding tanks. Unfertilized embryos were identified using a microscope and removed to select only viable ones for the exposure. Twenty embryos were transferred into 25 mL Pyrex beakers in a random fashion with 10 mL ERM to comprise the experimental treatments (ERM control, 25–5000 µg/L SMX). Four independent experiments were conducted with five to eight replicates per experimental group to assess survival, deformities, and hatchability of embryos. The EVOS™ Auto Imaging System (Thermo Fisher Scientific, Waltham, MA, USA) was used to visualize embryos. Deformities recorded included the presence of yolk sac edema, pericardial effusion, and kinked tails. Zebrafish were exposed continuously for seven days, and the exposure media was prepared and renewed on daily basis to maintain a constant exposure concentration.

### 2.4. Mitochondrial Bioenergetics

For the assessment of mitochondrial bioenergetics, 6 hpf zebrafish embryos were transferred into experimental beakers with 10 embryos each. Exposure treatments included ERM, 2, 20, 200, and 2000 µg/L SMX. Four replicate beakers were maintained for each exposure condition and control group. Following a 48-h exposure to SMX, a single surviving embryo from each of the beakers (*n* = 4/treatment, beaker was considered the biological replicate) was selected for the assessment of oxygen consumption rates (OCR) using the Seahorse XFe24 Extracellular Flux Analyzer (Agilent Technologies, Santa Clara, CA, USA) [25,26]. The Wave Desktop Software v 2.6 (Agilent Technologies) was used to export data to GraphPad PRISM v9.4 (La Jolla, CA, USA). Mitochondrial bioenergetics assessments included basal and maximal respiration, ATP-linked respiration, and non-mitochondrial respiration (User Guide Kit 103015-100, Agilent).

### 2.5. Reactive Oxygen Species (ROS)

The level of ROS was measured according to previously published methods [5]. Briefly, embryos were obtained and handled (as per methods outlined in Section 2.2 and Section 2.3) and exposed to one of ERM, 25, 100, and 500 µg/L SMX (*n* = 5 beakers per experimental group). Each beaker contained ~20 embryos. The experiment was conducted for 7 days with a daily media change to renew the chemical. At 7 days post fertilization (dpf), ROS levels in zebrafish larvae were measured using H_2_-DCFDA and a multi-detection microplate reader (New-Synergy 4, Bio-Tek, Singapore). Total protein was determined for each sample using a BCA assay (Thermo Fisher Scientific). ROS levels were expressed as normalized signal intensity/(μg/mL) protein.

### 2.6. Visual Motor Response Test (VMR)

Dark photokinesis response in zebrafish larvae was measured after exposure to SMX (0, 25, 50,100, 200, 500, 1000, 2500, and 5000 µg/L) using the VMR test. Five replicate beakers (15 embryos per beaker) were prepared for all treatments. Eight independent trials were performed to assess the potential effects of SMX on developing larvae behavior. In mid-afternoon (~2:00–3:00 p.m.), normally developed zebrafish larvae (*n* = 17–33 individuals/treatment) were placed into a 96-well plate. Activities of larvae were tracked using DanioVision™ (Leesburg, VA, USA). The assay followed our established protocol [25,26]. All trial data were merged into a single graph, and the total distance covered represented the level of locomotor activity.

### 2.7. Anxiety Test

Embryo and larval exposures proceeded as per Section 2.6. Zebrafish larvae (at 7 dpf) were transferred into a 12-well plate with ERM. Twenty trials were conducted with *n* = 13–36 fish per treatment for the light/dark preference test (LDPT). A company-manufactured cover was placed over the plate to create light and dark zones in each well. Poorly tracked larvae were excluded from analysis and data from twenty distinct runs were blended into a single graph to reflect all runs. Buspirone hydrochloride at 60 µM (CAS number: 33386-08-2, Sigma-Aldrich, St. Louis, MO, USA) was used as a positive control in this assay since it is an anxiolytic compound that has been validated in the LDPT with zebrafish larvae [27]. The assay methodology is described in detail in our previous publication [5].

### 2.8. Gene Expression Analysis

Zebrafish larvae at 6 hpf were exposed to either ERM or one of several concentrations of SMX (0, 25, 50, 100, 200, 500 µg/L) for gene expression analysis over seven days. Eleven to twelve fish from a single beaker were pooled in a tube to make one biological replication. To avoid RNA degradation, samples were subjected to liquid nitrogen and stored in a −80 °C ultra-freezer prior to RNA extraction. Nucleic acids were extracted using the TRIzol^®^ Reagent (Thermo Fisher, Waltham, MA, USA), and pellets were reconstituted in DNase- and RNase-free water. Utilizing the RNA-6000 nano kit on 2100- Bioanalyzer, RNA integrity was assessed (Agilent Technologies, CA, USA). The mean RNA integrity number (RIN) value for the samples was 8.87 ± 0.66. TURBO DNA free™ Kit was used to remove genomic DNA (Thermo Fisher Scientific). The iScript^TM^ cDNA (Bio-Rad, Hercules, CA, USA) was used to create the cDNA using 750 ng of RNA. The no reverse transcriptase (NRT) controls were created using five randomly chosen RNA samples in the same manner as above, but without the enzyme. One no-template control (NTC) without RNA template was also included in the plate. qPCR plates were prepared following the protocol: 0.8 µL of forward and reverse primers (approx. 100–200 nM), 3.33 µL (cDNA), and 5.025 µL (SsoFast™- EvaGreen^®^ -Supermix solution). The CFX Connect™ RT- PCR Detection System (Bio-Rad) was used to collect data as per previously published methods [28]. Two technical replicates were used to measure each biological replicate. Two housekeeping genes were used to standardize target expression: ribosomal subunit 18 (*rps18*) and beta-actin (*β-actin*) (M-value = 0.82, CV = 0.27). The average expression stability (M-value) of the reference genes is determined by GeNorm analysis (CFX Manger 3.1, BioRad). An individual reference gene is tested against the other reference genes in a pairwise variation that serially excludes the least stable genes from the analysis. The most stable reference genes (or combination) exhibit the lowest M-values. Less than M = 1.0 is considered relatively stable. CFX Manager^TM^ software (v3.1) was used to obtain normalized expression values for each target gene, and the relative ΔΔCq technique based on BioRad software was applied.

### 2.9. Acridine Orange Staining/Apoptosis Assay

The effect of SMX treatment on zebrafish apoptosis was assessed using the nucleic acid-selective staining technique known as acridine orange dye [29]. Briefly, 7 dpf larvae from each experimental group were randomly selected (*n* = 15) and washed with ERM after exposure to different concentrations of SMX (ERM, 25, 100, and 500 µg/L). After extensive washing, larvae were then placed into a 96-well plate with a 2 µg/mL acridine orange solution and stained for 30 min at room temperature without illumination. After washing with ERM (5 times for 30 s), apoptotic cells were visualized with an EVOS™ FL (Thermo Fisher Scientific, USA) using a GFP filter at 10× magnification. Fluorescence patches of vivid green color denoted apoptotic cells. The fluorescence intensity was quantified using the histogram tool of the Image J software v1.53t (http://rsbweb.nih.gov/ij/) (accessed on 15 August 2022).

### 2.10. Statistical Analysis

Data are presented as mean ± standard deviation (SD), and all data sets were statistically analyzed and graphed using GraphPad PRISM v9.4 (La Jolla, CA, USA). Kruskal-Wallis followed by Dunn’s multiple comparison test was used to assess deformities, survival, and hatch rate data. Data related to ROS, apoptosis, and transcript levels were assessed for normality by applying a Shapiro-Wilk test. Differences in group means were compared to the ERM and were analyzed using a one-way analysis of variance (ANOVA) followed by Dunnett’s multiple comparisons test. For locomotor activity and endpoints of anxiety, one-way ANOVA followed by Holm-Sídak post hoc test was used. For all analyses, the significance difference criterion was *p* < 0.05.

## 3. Results

### 3.1. Survival, Morphological Malformations, and Hatchability

**Figure 1:**
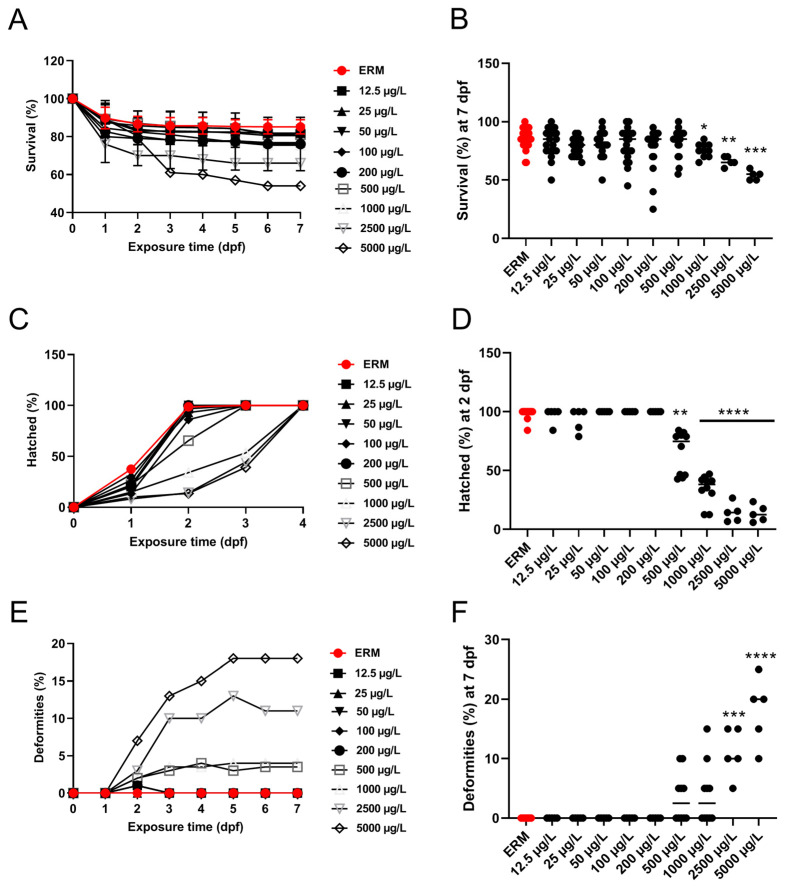
Percentage survival, deformities, and hatch rate of zebrafish exposed to ERM (control), 12.5, 25, 50, 100, 200, 500, 1000, 2500, and 5000 µg/L of sulfamethoxazole over time (**A**,**C**,**E**). Percent survival and deformities at 7 dpf and hatch rate at 2 dpf of zebrafish embryos ((**B**,**D**,**F**), respectively). Four separate experiments were performed, and data were merged into single graphs for all three parameters. The horizontal line represents the group’s median value (Kruskal Wallis followed by Dunn’s multiple comparison test, * *p* < 0.05, ** *p* < 0.01, *** *p* < 0.001, **** *p* < 0.0001).

**Figure 2:**
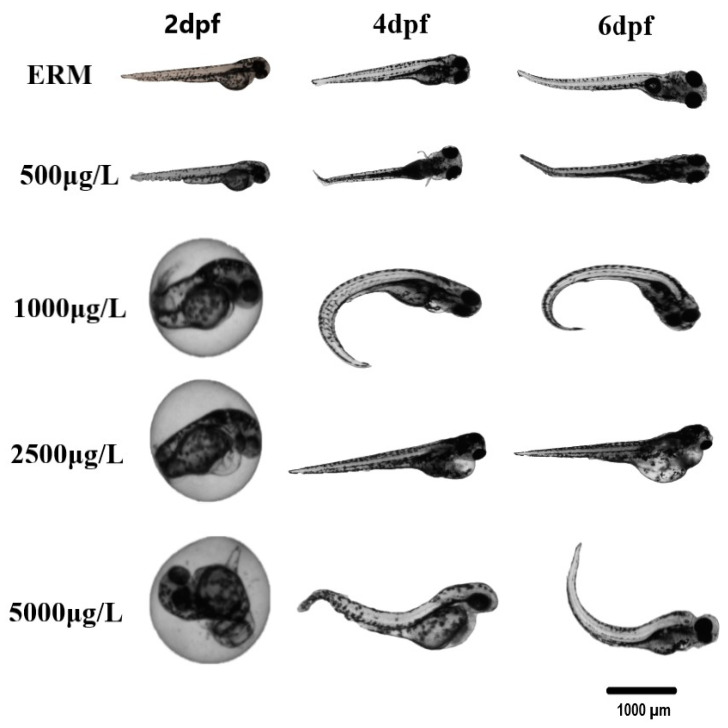
Selected photomicrographs of morphological deformities observed in zebrafish embryos/larvae at 2, 4, and 6 dpf after being exposed to one concentration of 500, 1000, 2500, and 5000 µg/L sulfamethoxazole and ERM (control). The scale bar is 1000 µm. Most of the deformities/malformations were observed at higher concentrations tested. Predominant deformities were yolk sac edema, pericardial effusion, bent tail, and curved spine.

In four different trials, the percentage of zebrafish larvae that survived each trial was noted, and daily images of malformations were obtained. Data were merged into a single representative graph. The survival rate of zebrafish larvae was significantly affected by SMX exposure (H = 35.90, *p* < 0.0001) (Figure 1A). At 7 dpf, a statistically significant increase in mortality at concentrations ranging from 1000 to 5000 μg/L was observed (Figure 1B). There was approximately a 30–40% mortality rate by the end of the exposure at the highest concentration tested (5000 μg/L). A statistically significant difference in the hatch rate at 2 dpf among groups was recorded (H = 63.68, *p* < 0.0001) (Figure 1D). The control group (ERM) and environmentally relevant concentrations of SMX showed no apparent deformities in larvae (<2% of all tested animals). However, there was a significant increase (H = 49.58, *p* < 0.0001) in the occurrence of malformations at 7 dpf following exposure to higher concentrations (2500 and 5000 μg/L) (Figure 1E,F). The most common deformities observed in higher concentrations tested were yolk sac edema, pericardial effusion, deformed tail, and curved spine (Figure 2).

### 3.2. Mitochondrial Bioenergetics

The oxygen consumption rate (OCR) of embryos (at 54 h of age) exposed to SMX was determined following a two-day exposure (Supplementary Figure S1A–E). No significant change was observed for all the parameters tested: basal respiration (F_(4,15)_ = 1.57, *p* = 0.23), ATP-linked respiration (F_(4,15)_ = 1.81, *p* = 0.18), maximal respiration (F_(4,15)_ = 0.74, *p* = 0.68), and non-mitochondrial respiration (F_(4,15)_ = 0.34, *p* = 0.85).

### 3.3. Reactive Oxygen Species (ROS)

Zebrafish embryos were exposed to either 25, 100, or 500 µg/L SMX or ERM for seven days. None of the treatment groups showed differences in the level of ROS compared to ERM (F_(3,16)_ = 0.79, *p* = 0.51) (Supplementary Figure S2).

### 3.4. Gene Expression (RT-PCR) Analysis

**Figure 3:**
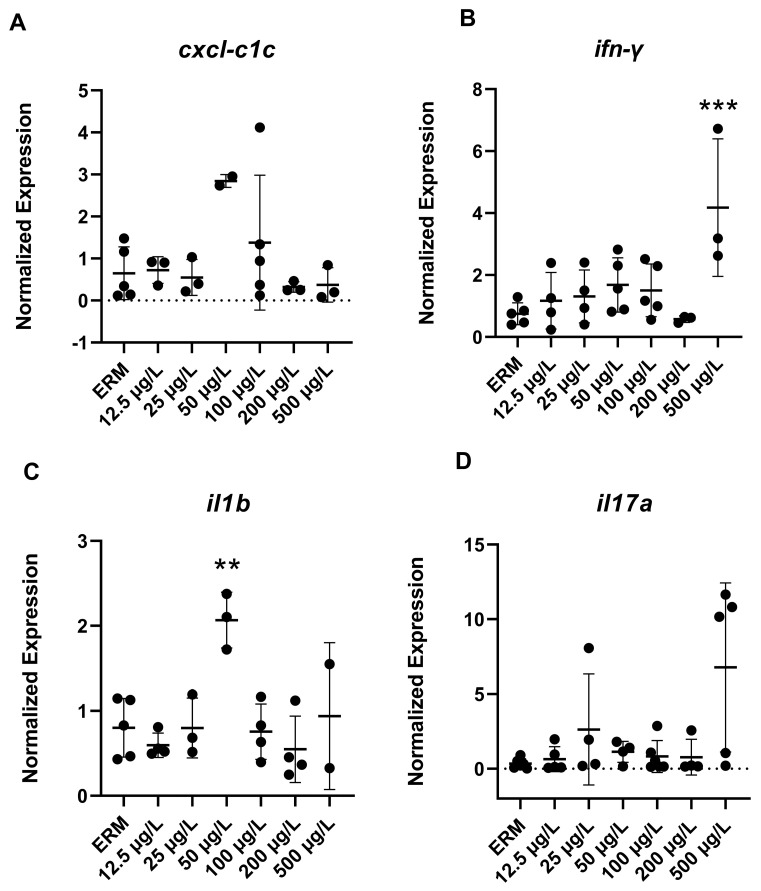
Transcript levels of immune system: (**A**) chemokine *cxcl-c1c*; (**B**) interferon gamma, *ifn-γ*; (**C**) Interleukin-1beta, *il-1b*; (**D**) Interleukin-17A, *il-17* in 7 dpf zebrafish larvae exposed to sulfamethoxazole. Data are represented as mean (±SD). One-way ANOVA followed by Dunnett’s post-hoc test, ** *p* ≤ 0.01, *** *p* ≤ 0.001, *n* = 3–5 biological replicates/treatment.

**Figure 4:**
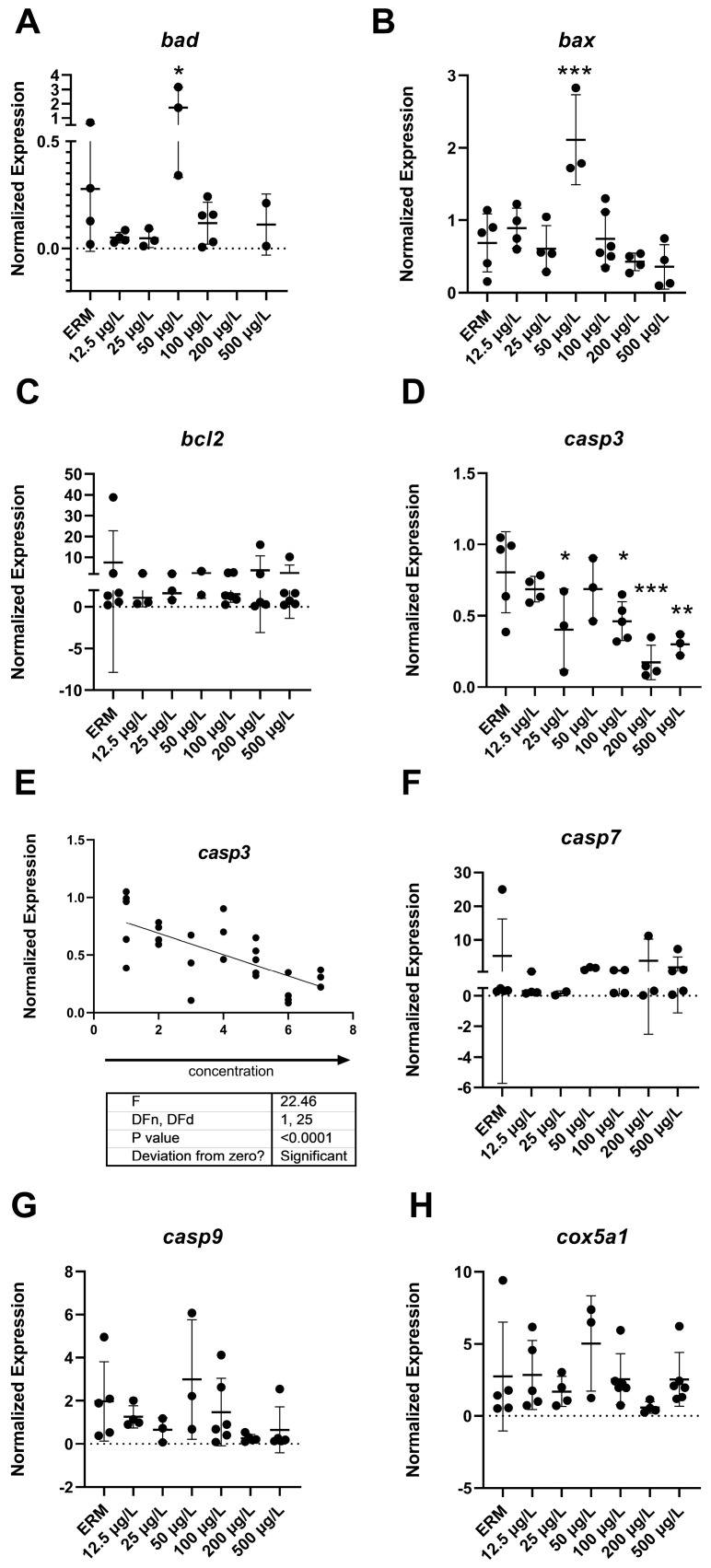
Transcript levels of apoptosis: (**A**) Bcl2 associated agonist of cell death, *bad*; (**B**) Bcl2 associated x, apoptosis regulator, *bax*; (**C**) Bcl2 apoptosis regulator, *bcl2*; (**D**) Caspase 3a, *casp3a* (**E**) Linear regression of caspase 3a, *casp3a* (**F**); Caspase 7, *casp7* (**G**); Caspase 9, *casp9* (**H**); Cytochrome c oxidase subunit 5Aa, *cox5a1* in 7 dpf zebrafish larvae exposed to sulfamethoxazole. Data represented as mean (±SD). One-way ANOVA followed by Dunnett’s post-hoc test, * *p* ≤ 0.05, ** *p* ≤ 0.01, *** *p* ≤ 0.001, *n* = 3–5 biological replicates/treatment.

**Figure 5:**
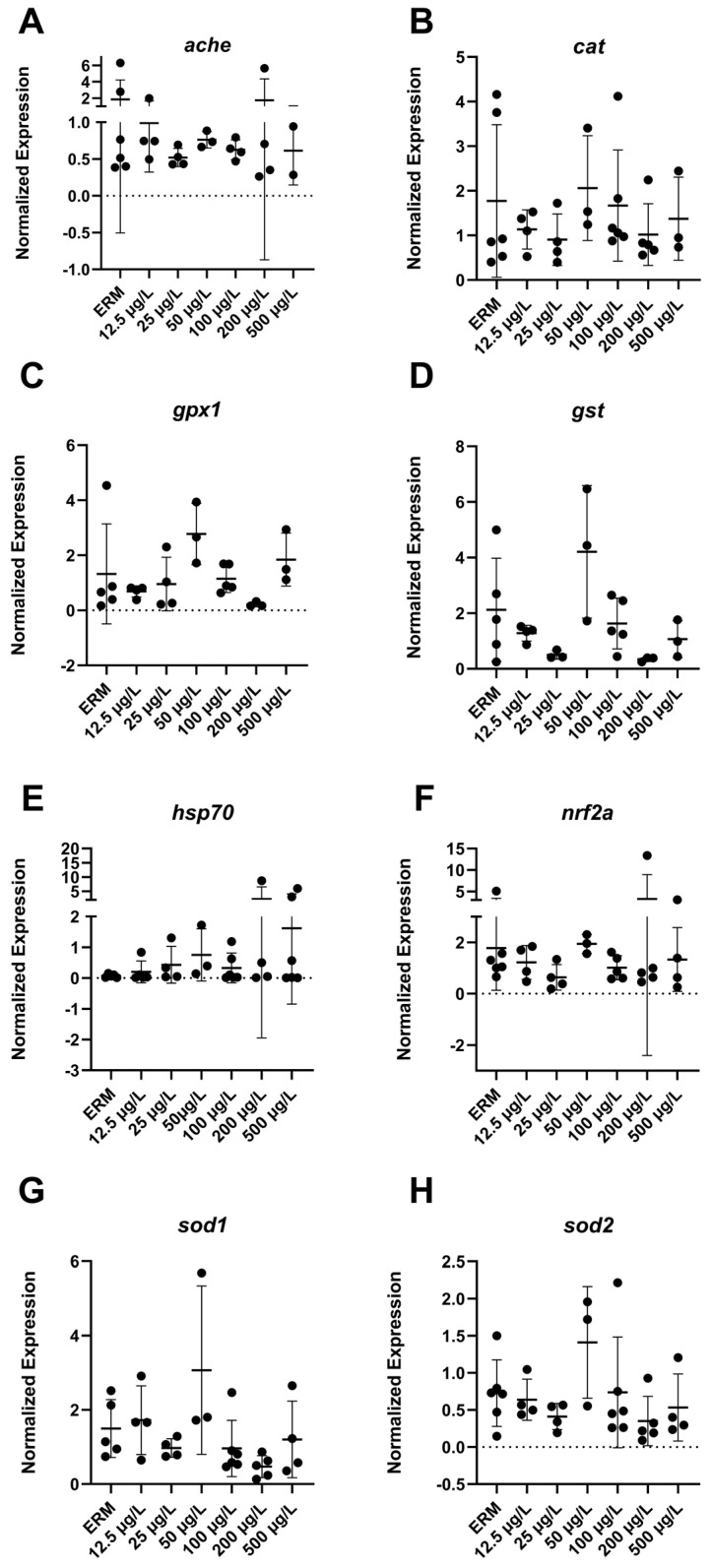
Transcript levels of oxidative stress: (**A**) acetylcholinesterase, *ache*; (**B**) catalase, *cat*; (**C**) glutathione peroxidase 1, *gpx1*; (**D**) glutathione S-transferase, *gst* (**E**) heat shock protein 70, *hsp70*; (**F**) nuclear factor erythroid 2-related factor 2, *nrf2a*; (**G**) superoxide dismutase 1, *sod1*; (**H**) superoxide dismutase 2, *sod2* in 7 dpf zebrafish larvae exposed to sulfamethoxazole. Data represented as mean (±SD). One-way ANOVA followed by Dunnett’s post-hoc test, *p* > 0.05, *n* = 3–5 biological replicates/treatment.

The mRNA levels of a wide number of transcripts related to oxidative stress, apoptosis, and the immune system were measured to determine the potential effects of SMX on developing zebrafish larvae. Transcript levels of immune-related gene *ifn-γ* (F_(6,22)_ = 4.8, *p* = 0.0029) (Figure 3B) in larvae showed increased expression with exposure to 500 µg/L SMX. In addition, *IL-1β* was upregulated (F_(6,18)_ = 6.07, *p* = 0.0013) in zebrafish at 50 µg/L exposure group compared to ERM (Figure 3C). Interestingly, transcript levels for apoptosis-related gene *casp3a* (F_(6,20)_ = 5.84 *p* = 0.0012) (Figure 4D–E) were reduced in larvae in a concentration-dependent manner as compared to the ERM while transcript levels for *bad* (F_(5,15)_ = 4.78, *p* = 0.0082) (Figure 4A) and *bax* (F_(6,23)_ = 8.69, *p* < 0.0001) (Figure 4B) were upregulated in fish exposed to 50 µg/L. SMX exerted no effect on oxidative stress-related transcripts tested in the study (*p* > 0.05) (Figure 5).

### 3.5. Apoptosis Analysis

**Figure 6:**
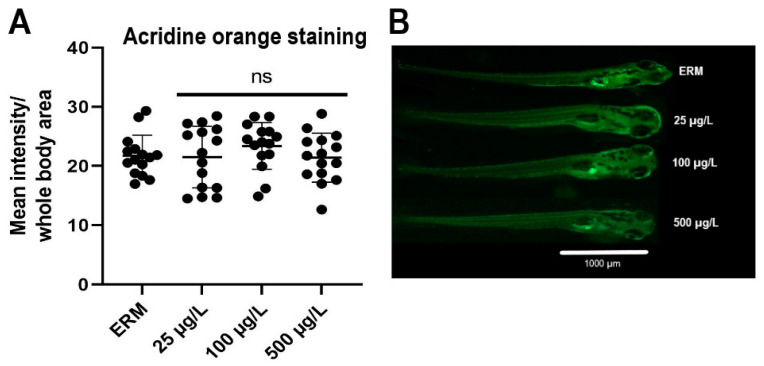
(**A**) Fluorescence intensity (quantified by using the histogram tool of the Image J software) in zebrafish larvae exposed to ERM (control), 25, 100, and 500 µg/L sulfamethoxazole at 7 dpf. Data represented as mean (±SD) (one-way ANOVA followed by Dunnett’s post-hoc test, *p* > 0.05, *n* = 15 larvae per treatment). (**B**) Representative photomicrographs of zebrafish larvae stained with acridine orange (AO) dye after being exposed to ERM (control), 25, 100, and 500 µg/L sulfamethoxazole for 7 days. The scale bar is 1000 µm. The abbreviation ns indicates not significant.

The exposure effect of SMX on zebrafish embryonic apoptosis was determined at 7 dpf using acridine orange staining (Figure 6A). No significant difference was detected for the apoptotic levels in treatment groups compared to ERM (F_(3,56)_ = 0.72, *p* = 0.55) (Figure 6B).

### 3.6. Visual Motor Response Test (VMR) and Light-Dark Preference Test (LDPT)

**Figure 7:**
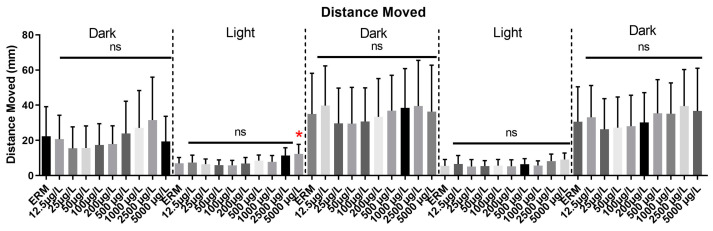
Visual Motor Response Test (VMR) for zebrafish larvae exposed to ERM (control), 12.5, 25, 50, 100, 200, 500, 1000, 2500, and 5000 µg/L concentrations of sulfamethoxazole at 7 dpf. Each light and dark cycle shows a 10-min interval. Data from eight independent trials are merged as a single graph. One-way ANOVA followed by Holm’Šídák’s multiple comparisons test, ns = non-significant, * *p* ≤ 0.05, *n* = 17–32 larvae per treatment/experiment).

Eight independent trials of the VMR test were conducted and data from all trials were pooled to generate a single graph (Figure 7). Combined distance moved differed among experimental groups from the control in both light and dark zones (F_(49,450)_ = 6.8, *p* < 0.0001). Analysis of each light and dark cycle separately showed an increase in locomotor activity for zebrafish treated with the 5000 µg/L group in the first light cycle. However, in subsequent dark and light cycles, there was no significant effect on locomotor activity in all treatments tested.

**Figure 8:**
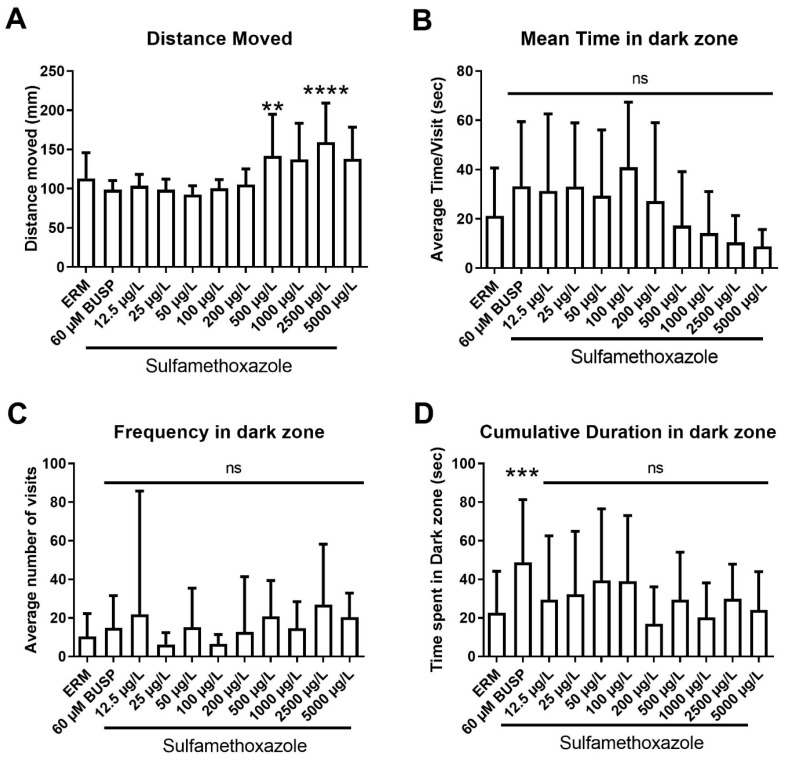
Light-dark preference test (LDPT) showing anxiolytic behavior of zebrafish exposed to ERM (control), 12.5, 25, 50, 100, 200, 500, 1000, 2500, and 5000 µg/L of sulfamethoxazole at 7 dpf. (**A**) Total distance moved (mm); (**B**) mean time in dark zone; (**C**) frequency in dark zone; (**D**) cumulative duration in dark zone. (One-way ANOVA followed by Holm-Šídák’s multiple comparisons test, ns = non-significant, ** *p* ≤ 0.01, *** *p* ≤ 0.001, **** *p* ≤ 0.0001, *n* = 13–32 larvae per treatment).

We also assessed anxiolytic behaviors in larval zebrafish using a light-dark preference test. A significant increase in total distance traveled in larval fish (F _(10,213)_ = 8.44, *p* < 0.0001) (Figure 8A) was observed with 500 and 2500 µg/L SMX. Mean time spent in the dark zone total (F _(10,200)_ = 3.81, *p* < 0.0001) (Figure 8B) showed a decreasing trend with respect to concentration. Frequency in the dark zone (F _(10,213)_ = 1.42, *p* = 0.17) (Figure 8C) did not differ between experimental groups. Cumulative time spent in the dark zone (F _(10,213)_ = 2.98, *p* = 0.0015) (Figure 8D) revealed that only the positive control buspirone enhanced the mean time of zebrafish in the dark zone and there was no effect of SMX. Taken together, there was a subtle effect of SMX in larval fish, and a slight increase in locomotor activity with the highest concentrations tested.

## 4. Discussion

Antibiotics are considered emerging contaminants and are ubiquitously present in aquatic environments because of their widespread use in human and animal medicine. Several biomarkers have been used to quantify the toxicity of these chemicals in a range of aquatic animals. However, the toxicity of several antibiotics in aquatic organisms has not been fully assessed and continued surveillance is needed for risk assessment. SMX has been reported to be as high as 16 mg/L (16,000 µg/L) in some aquatic environments [13], raising concerns of its toxicity to exposed organisms. Exposure to SMX has been reported to induce immunosuppression, antibiotic resistance, biochemical responses, and histopathological changes in aquatic organisms [16,20,30]. The current study determined the exposure effects of a broad range of SMX concentrations to developing zebrafish, an ideal indexical organism for ecotoxicological studies. Our results inform on potential sublethal effects related to survival, development, gene expression, oxidative stress, mitochondrial metabolism, and behavioral performance of zebrafish embryos under exposure to SMX.

We observed that SMX affected survival, hatchability, and caused malformations in developing zebrafish. SMX exposure at concentrations of 2500 and 5000 µg/L caused gross morphological abnormalities, which frequently appeared as early as 2 dpf. The most common deformities observed were yolk sac edema, pericardial effusion, bent tail, and curved spine. A study conducted to assess the effects of three types of sulfonamides: sulfamethoxazole, sulfadiazine, and sulfadimidine at (1–1000 µg/L) revealed that SMX induced deformities in zebrafish, which included yolk sac edema, hemagglutination, and axial malformation [31]. This same study also showed an overall decline in the hatchability with increased exposure concentrations. No differences were noted for low concentrations (1 µg/L and 100 µg/L) for all three sulfonamides. A substantial decrease in hatching rate was observed at a higher concentration of 1000 µg/L SMX and hatchability was reported to be lower compared to the control group for SMX exposure (by approximately 11.5%). Our data agree with past investigations and acute toxicity is not observed until concentrations reach 500 µg/L exposure or more. In another study, decreased body length and delayed hatching were observed in zebrafish embryos exposed to 100 µg/L SMX [17]. We observed effects on the hatch rate in zebrafish embryos exposed to 500 µg/L, but not 100 µg/L. In another study, zebrafish embryos exposed to sulfamethazine (0.2–2000 µg/L) presented a reduced embryo hatching rate at any given concentration between 58 and 96 hpf [30]. Spinal curvature and edema were also reported as the two primary types of malformations induced by the antibiotic. Thus, exposure to SMX is responsible for a delay in hatchability in zebrafish embryos.

The hatchability delay and malformations observed in the current study may be explained by the chemical actions of SMX [31] and several mechanisms may underlie developmental effects. Epiboly is observed in zebrafish embryos during the gastrula stage. Sulfonamides are an example of an exogenous antibiotic that can harm cells in the pre-epiboly stage as well as inhibit embryonic growth. According to a study, the lack of epiboly function brought on by sulfonamides can impede the developmental process of the anterior-posterior body axis. For instance, a neural tube deficiency may have led to the tail flexion observed in the current study [32]. The appearance of yolk sac edema is also linked with an unusual loss of epiboly action [33], which was also observed in some fish in the current study. Based on other studies [34,35], sulfonamides may react with cytoplasmic receptors before moving to the nuclei, exerting a negative effect on embryonic development. Another mechanism for developmental effects may also involve the thyroid hormone system. As thyroglobulin (Tg) disruptors, sulfonamides cause dilatation and degranulation of rough endoplasmic reticulum [31]; these modifications could result in low levels of Tg secretion. The development of organs and the central nervous system would be impacted by subsequent hypothyroidism. Lastly, the delayed hatchability and malformations of zebrafish embryos may be explained by the chemical actions of SMX specifically impairing cell division by regulating folate metabolism [31].

Abnormal oxidative respiration is indicative of oxidative stress and mitochondrial dysfunction. In our study, SMX exposure did not affect the oxygen consumption rates of embryos, nor did it induce ROS in larvae at the tested concentrations. In line with this, we did not observe a significant change in oxidative stress-related transcripts (*cat*, *gpx1*, *gst*, *hsp70*, *nrf2a*, *sod1* and *sod2*). In another study, changes in mitochondrial function were observed at 73 μg/L concentration as compared to 14.7 mg/L of erythromycin, confirming the dysfunction of mitochondria at higher dosages of antibiotics [36]. Aquatic studies have utilized biomarkers of oxidative stress frequently. Investigating diverse endpoints connected to oxidative stress is crucial, as an organism’s reaction to a xenobiotic or tissue-specific stressor can vary. To cope with ROS produced by oxygen-involved natural and external stresses, organisms often activate several enzymatic systems. Reactive oxygen species have a high intrinsic reactivity, which makes them potentially harmful to cells. As a result, anti-oxidant defense systems may not be sufficient to prevent the emergence of oxidative stress. For the exposure time and SMX concentrations investigated here, ROS production was not a significant mechanism as SMX did not induce oxidative stress or mitochondrial dysfunction in early tagged embryos. A lack of antioxidant defense in response to antibiotic exposure has been reported previously in zebrafish. Exposure to SMX at 50–500 µg/L for 14 days did not alter the activity of antioxidant enzymes in zebrafish [37]. Another study reported evidence of oxidative stress in the brain of *Cyprinus carpio* exposed to 200 µg/L of SMX in a 28-day chronic exposure [20]. Thus, evidence can be mixed for the support of oxidative stress responses in aquatic organisms exposed to sulfonamides. These differences may be attributed to different fish species and life stage of the fish (larval or adult fish), as well as concentration, exposure duration, and time point evaluated.

A series of immune-related transcripts were measured in zebrafish to assess the immune response of zebrafish embryos, comprising inflammatory cytokines such as *IL-1β*, *ifn*, *il17a* and *cxcl-c1c*. These genes were chosen because antibiotic exposure has been reported to affect the cytokines of fish [38]. Our results indicated up-regulation of proinflammatory cytokines *ifn*-γ and *IL-1β*, generating insight into host exposure to antibiotics. In this study, SMX appears to induce inflammation in healthy zebrafish embryos during early developmental stages. The phenomenon of inflammation is dynamic in nature. Neutrophils in the body’s immune response generate several cytokines along with increased ROS when fish are infected by pathogens or harmed by pollutants [39]. Increases in *IL-1β* may result in the generation of lipid mediators, proteases, and ROS, and *IL-1β* is regarded as a key proinflammatory cytokine. Moreover, *ifnγ* is known to facilitate inflammation by inducing macrophages to produce TNF-α and *IL-1β* [40]. In the current study, the proinflammatory cytokine *IL-1β* was overexpressed in larvae following 50 µg/L SMX exposure, indicating environmental levels of antibiotics may induce inflammation in healthy zebrafish larvae. This is in line with previous studies showing that fish, including zebrafish and Nile tilapia, are sensitive to low doses of SMX exposure in terms of proinflammatory cytokines production [3]. This study broadens our understanding of the immune system in early life stages of fish and suggests antibiotic exposure at environmental levels may cause immunological disorders.

Apoptosis is a crucial cellular process for normal embryonic development. It is often measured to determine how antibiotics may affect the normal apoptosis rate in developing zebrafish larvae. Additionally, several transcripts related to apoptosis were evaluated in our study, including *bad*, *bax*, *bcl2*, *casp3a*, *casp7*, and *casp9*. There was a concentration-dependent decrease in *casp3a* expression with SMX exposure in zebrafish. Upregulation of *bad* and *bax* transcripts with SMX at an environmentally relevant concentration (50 µg/L) was also observed. The Bcl-2 family comprises both pro-apoptotic proteins, including (BAX, BAD), and anti-apoptotic proteins (Bcl2). When exposed to pollutants, *p53* transcription directly targets the *bax* genes, which are located on the mitochondrial outer membrane and play a key role in the commencement of apoptotic damage. The *p53-Bax* cascade primarily activates the intrinsic apoptosis pathway, which subsequently causes cell death via the mitochondria-dependent pathway [41]. In line with this mechanism, higher expression of *bad* and *bax* in exposed zebrafish may indicate that SMX exposure increases apoptotic signaling in fish. Similar results were also reported in Nile tilapia [3]. The increased inflammation at lower dosages of SMX also demonstrates the negative influences of SMX exposure on fish health.

The effector protein known as caspase 3 is essential for both endogenous (the mitochondrial) and exogenous (the death receptor) pathways of apoptosis. Additionally, it is the enzyme that controls synaptic activation and regulates the process of neurogenesis in developing larvae [42]. Transcripts for *casp3a* were decreased in relative abundance after SMX exposure in a dose-dependent manner, revealing disturbances in the normal apoptotic process, which is crucial for the development of embryos and repairing of damaged tissues. This may lead to developmental malformations in zebrafish embryos. In embryonic development, the apoptotic process mainly occurs during the eradication of redundant cellular material, which is essential for the correct morphogenesis of tissues and organs, in addition to maintaining tissue homeostasis throughout the life of the cell [43]. The expression of *casp3* is essential for development; knockout mice for caspase-3 were born infrequently and died within a short period of time [44]. Caspase-3-dependent apoptosis is essential for normal development of zebrafish embryos and aids in stress tolerance during zebrafish developmental period. An earlier study carried out by Yamashita et al. [45] reported on specific effects of caspase-3 repression (induced by microinjection of antisense MO (morpholino-oligonucleotide) in zebrafish embryos. At a lower dosage of caspase-3-MO, zebrafish embryos showed a slightly dorsalized appearance. At a higher dose of caspase-3-MO, the phenomenon of epiboly was arrested at 8–12 h post fertilization indicating that caspase deficiency blocked process of embryogenesis in targeted zebrafish embryos [45]. Lastly, components of the apoptotic pathway, including caspases, are essential for other physiological functions in a wide range of cell types, including neurons. A study conducted by Campbell et al. [46] reported that caspase activation is a key promoter of axon remodeling in the zebrafish embryos central nervous system, and decreased caspase-3 activity resulted in limited arbour expansion and synaptogenesis. Taken together, inhibition of caspase activity early in development can subsequently lead to developmental defects and behavioral abnormalities.

Our findings of altered apoptotic signaling in zebrafish exposed to SMX are supported by other studies on antibiotic-induced toxicity in fish. For example, increased expression of *bax* and *bcl-2* has been noted in the brain of *Cyprinus carpio* following exposure to cypermethrin [47]. Moreover, Xi et al. (2019) also reported that the antibiotic norfloxacin triggered apoptosis in zebrafish embryos [44]. We did not detect a change in apoptosis based upon AO staining; however, there may be a more subtle response of SMX and low-level effects on the transcriptome that is not fully captured with AO staining. Subsequently, a longer exposure may be required to induce apoptosis following up-regulation in the apoptosis-related transcripts at environmentally relevant concentrations. Considering that SMX can be persistent in the environment, apoptosis may be observed with sub-chronic exposures.

Zebrafish larvae are often used to study the neurotoxicity of aquatic pollutants. Several antibiotics have a negative impact on the locomotor activity of zebrafish [48]. Here, the visual motor response test revealed hyperactivity in the first light period at 5000 µg/L. However, that concentration is considerably higher than environmentally relevant levels of SMX reported globally. According to previous reports, SMX raises plasmatic bilirubin levels, causing kernicterus and related brain damage [49]. This may lead to behavioral deficits. Moreover, SMX exposure has been shown to induce neurotoxicity in grass carp by changing the permeability of the blood brain barrier and down-regulating tight junction proteins (occluding, claudins), thus causing abnormal behavior, histopathological changes, and ultrastructural damage (nerve cell damage and synapse reduction) [50]. Wang et al. (2014) studied the effect of six selected antibiotics on zebrafish behavior and data revealed that at 6.26 mg/L, zebrafish larvae showed increased movement and signs of neurotoxicity [51]. In our study, some hyperactivity was also observed in fish undergoing the light-dark preference test. We observed that responses in zebrafish larvae were affected by SMX in terms of total distance moved, but there was no evidence of anxiety in the larvae with SMX exposure. Almeida et al. (2019) noted anxiolytic behaviors in zebrafish larvae following exposure to the antibiotic oxytetracycline [52]. Their results reported that 10,000 µg/L exposure induced hyperactivity, changed feeding patterns, and reduced antioxidant enzymes in zebrafish larvae after long-term exposure. Taken together, SMX may pose a minimal risk to zebrafish for neurotoxicity at environmental concentrations (as no change in acetylcholinesterase expression was observed). However, further investigations into the hyperactivity response should be conducted in other fish species following exposure to antibiotics.

## 5. Conclusions

To conclude, SMX exerted acute toxicity to developing embryos and larvae of zebrafish at higher concentrations. SMX caused hatching delay, affected survivability, and induced deformities at higher concentrations tested. There was approximately 50% mortality at the end of the exposure at the highest concentration tested (5000 μg/L). The most prevalent deformities observed were edema of the yolk sac, pericardial effusion, bent tail, and curved spine. No changes were observed for endpoints related to oxidative stress and mitochondrial bioenergetics at concentrations tested in study. However, environmentally relevant concentrations of SMX disrupt the immune system by inducing *ifnγ* and *IL-1β* transcripts at 50 and 500 μg/L, respectively. SMX induces apoptosis by increasing *bad* and *bax* expression at 50 μg/L concentration.

The observed delay in hatching rate and malformations observed in zebrafish embryos may be associated with the inflammatory process due to an innate immune response of zebrafish larvae. Therefore, additional effort is needed to ensure that antibiotics are not overused and unnecessarily discharged into aquatic ecosystems. The current study reveals the potential ecological risks of antibiotics in the aquatic biome and provides baseline data for their safety and risk assessment.

## Data Availability

Not applicable.

## Supplementary Materials

The following supporting information can be downloaded at: https://www.mdpi.com/article/10.3390/toxics11020178/s1, Figure S1: Mitochondrial bioenergetics in 54 hpf zebrafish embryos. (A) Oxygen consumption rate (OCR); (B) basal respiration; (C) ATP-linked respiration; (D) maximal respiration; (E) non-mitochondrial respiration. Data are reported as mean ± SD (one-way ANOVA followed by Dunnett’s multiple comparisons test, *p* > 0.05). Figure S2: Reactive oxygen species (ROS) in zebrafish embryos exposed to sulfamethoxazole for 7 days expressed as relative fluorescence units (µg/mL protein). Horizonal line represents mean value of the group (±SD) (ANOVA followed by a Dunnett’s test, *n* = 5 per treatment); Table S1: Primers used for real-time PCR analysis [28,53,54,55,56,57,58,59,60,61,62,63,64,65,66,67,68,69].
