# Supplementary material for: Sulfamethoxazole (SMX) Alters Immune and Apoptotic Endpoints in Developing Zebrafish (Danio rerio)
Source: Toxics. 2023 Feb 14;11(2):178. doi: 10.3390/toxics11020178 (PMC9959310; doi:10.3390/toxics11020178)
Supplement: Supplementary file 1 [file toxics-11-00178-s001.zip › toxics-2180498-supplementary.pdf]

SUPPLEMENTAL DATA

Sulfamethoxazole (SMX) Alters Immune and Apoptotic Endpoints in Developing Zebrafish (*Danio rerio*)

Nazish Iftikhar [1,2], Isaac Konig [2,3], Cole English [2], Emma Ivantsova [2], Christopher L. Souders II [2], Imran Hashmi [1] and Christopher J. Martyniuk [2,4,*]

[1] Institute of Environmental Sciences and Engineering, School of Civil and Environmental Engineering, National University of Sciences and Technology, Sector H-12, Islamabad 44000, Pakistan
[2] Center for Environmental and Human Toxicology, Department of Physiological Sciences, College of Veterinary Medicine, University of Florida, Gainesville, FL 32611, USA
[3] Department of Chemistry, Federal University of Lavras (UFLA), Lavras 37203-202, Minas Gerais, Brazil
[4] UF Genetics Institute and Interdisciplinary Program in Biomedical Sciences Neuroscience, University of Florida, Gainesville, FL 32611, USA
* Correspondence: cmartyn@ufl.edu; Tel.: +1-352-294-4642; Fax: +1-352-392-4707

Supplemental Table S1. Primers used for real-time PCR analysis.

**Gene name**	**Gene Symbol**	**Forward (5' to 3')**	**Reverse (5' to 3')**	**Reference**
Acetylcholinesterase	*ache*	GCTAATGAGCAAAAGCATGTGGGC	TATCTGTGATGTTAAGCAGACGAGGCA	NM_131846.2
BCL2 Apoptosis Regulator	*bcl-2*	TCACTCGTTCAGACCCTCAT	ACGCTTTCCACGCACAT	Deng et al. 2009
BCL2 Associated Agonist Of Cell Death	*bad*	CAAGCCTGGATAAACAC	GGCAGATTGAAAGAAAG	Lu et al. 2011
BCL2 Associated X, Apoptosis Regulator	*bax*	GGCTATTTCAACCAGGGTTCC	TGCGAATCACCAATGCTGT	Liu et al., 2021
Beta-actin	*bactin*	CGAGCAGGAGATGGGAACC	CAACGGAAACGCTCATTGC	Wang et al. 2018
Caspase 3	*casp 3*	CCGCTGCCCATCACTA	ATCCTTTCACGACCATCT	Deng et al. 2009
Caspase 7	*casp 7*	TTCGAGTCCTGGTCGGAAGA	CCTTGCCTGCCATCCTGTAA	MG957999.1
Caspase 9	*casp 9*	ACCTCAATGGCCAGAACTGTC	TCCTCCAGCACACGATCAAG	Deng et al. 2009
Catalase	*cat*	CTCCTGATGTGGCCCGATAC	TCAGATGCCCGGCCATATTC	Sarkar et al., 2014
chemokine (C-X-C motif) ligand 18b<br>Glutathione peroxidase 1a	*Cxcl-C1c alias*<br>*gpx1a*	GGCATTCACACCCAAAGCG<br>CACCCTCTGTTTGCGTTCC	GCGAGCACGATTCACGAGAG<br>CTCTTTAATATCAGCATCA	Stockhammer et al., 2009<br>Wu et al., 2016
Heat shock protein 70	*hsp70*	GAAGACGGCATCTTTGAGGTGA	GGGCCCTCTTGTTCTGACTGAT	Hahn et al., 2014
Interferon Gamma<br>Interleukin 17	*ifng*<br>*il-17a*	GAATGGCTTGGCCGATACAGGATA<br>CGAGAGCCTGTATCCTAC	TCCTCCACCTTTGACTTGTCCATC<br>CGTAATCCTGGACCTCAA	Jin et al., 2010<br>Zhang et al., 2014

Interleukin 1b	*IL-1β*	TGGACTTCGCAGCACAAAATG	GTTCACTTCACGCTCTTGGATG	Watzke et al., 2007
Ribosomal 18s	*rps18*	TCGCTAGTTGGCATCGTTTATG	CGGAGGTTCGAAGACGATCA	Wang et al. 2018
Superoxide dismutase 1	*sod1 (Cu/Zn SOD)*	CAACACAAACGGCTGCATCA	TTTGCAACACCACTGGCATC	Sarkar et al., 2014
Superoxide dismutase 2	*sod2 (Mn SOD)*	AGCGTGACTTTGGCTCATTT	ATGAGACCTGTGGTCCCTTG	Sarkar et al., 2014
Glutathione S-transferase	*gst*	CTATACATGCGGCGAAGCT	GGCATTGCTCTGGACGAT	Mukhopadhyay et al., 2014
Nuclear factor erythroid 2-related factor 2	*nrf2a*	CCAGATCCAATCGTGGAGTTT	GAAGGATCCGTCTTCGGTTATG	Awoyemi et al., 2019

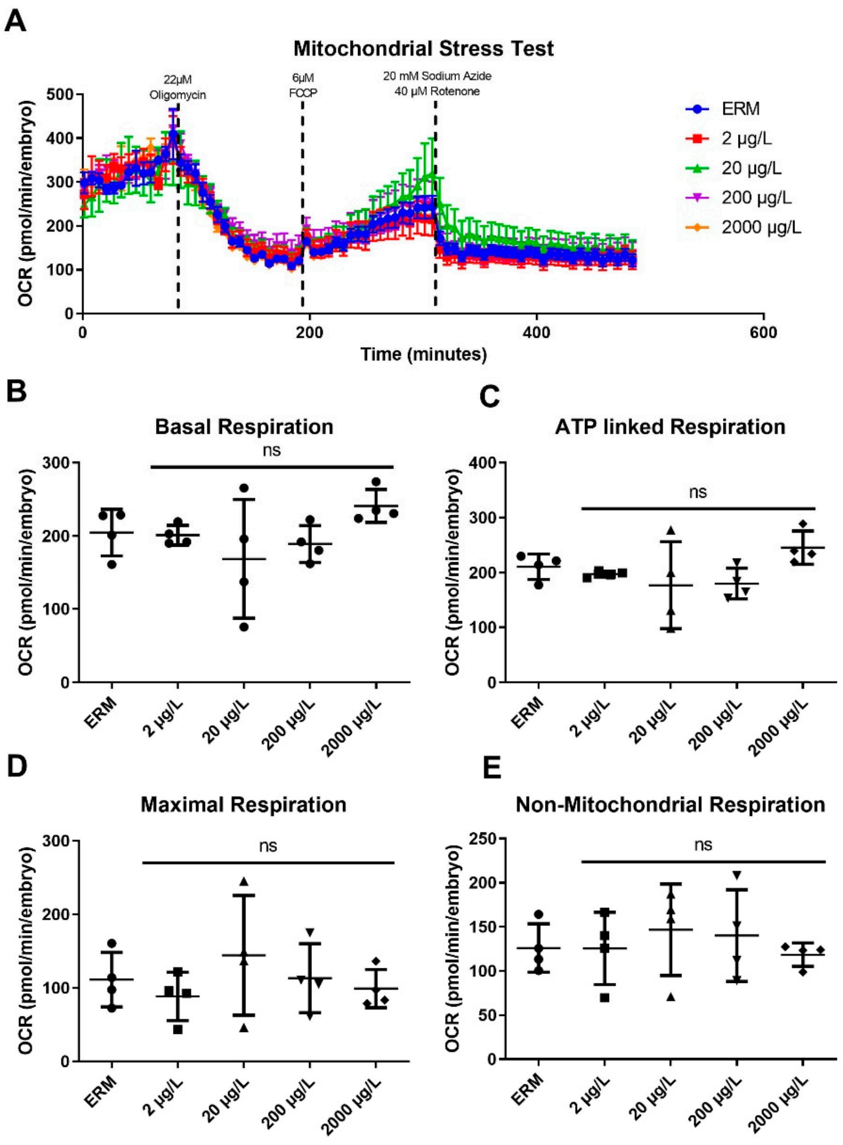

Supplemental Figure S1. Mitochondrial bioenergetics in 54 hpf zebrafish embryos. (A) Oxygen consumption rate (OCR); (B) basal respiration;

(C) ATP-linked respiration; (D) maximal respiration; (E) non-mitochondrial respiration. Data are reported as mean ± SD (one-way ANOVA followed by Dunnett's multiple comparisons test, $p > 0.05$).

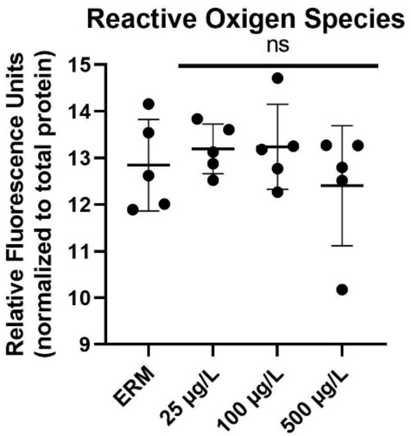

Supplemental Figure S2. Reactive oxygen species (ROS) in zebrafish embryos exposed to sulfamethoxazole for 7 days expressed as relative fluorescence units (µg/mL protein). Horizonal line represents mean value of the group (±SD) (ANOVA followed by a Dunnett's test, n=5 per treatment).

References

Awoyemi, O. M., Kumar, N., Schmitt, C., Subbiah, S., & Crago, J. (2019). Behavioral, molecular and physiological responses of embryo-larval zebrafish exposed to types I and II pyrethroids. Chemosphere, 219, 526-537.

Deng, J., Yu, L., Liu, C., Yu, K., Shi, X., Yeung, L.W.Y., Lam, P.K.S., Wu, R.S.S., Zhou, B., 2009. Hexabromocyclododecane-induced developmental toxicity and apoptosis in zebrafish embryos. Aquat. Toxicol. 93, 29-36.

Hahn, M.E., McArthur, A.G., Karchner, S.I., Franks, D.G., Jenny, M.J., Timme-Laragy, A.R., Stegeman, J.J., Woodin, B.R., Cipriano, M.J. and Linney, E., 2014. The transcriptional response to oxidative stress during vertebrate development: effects of tert-butylhydroquinone and 2, 3, 7, 8-tetrachlorodibenzo-p-dioxin. PloS one, 9(11), p.e113158.

Jin, Y., Chen, R., Liu, W. and Fu, Z., 2010. Effect of endocrine disrupting chemicals on the transcription of genes related to the innate immune system in the early developmental stage of zebrafish (Danio rerio). *Fish & shellfish immunology*, *28*(5-6), pp.854-861.

Liu, L., Wu, F.Y., Zhu, C.Y., Zou, H.Y., Kong, R.Q., Ma, Y.K., Su, D., Song, G.Q., Zhang, Y. and Liu, K.C., 2021. Involvement of dopamine signaling pathway in neurodevelopmental toxicity induced by isoniazid in zebrafish. Chemosphere, 265, p.129109.

Lu, J., Tsai, T., Choo, S., Yeh, S., Tang, R., Yang, A., Lee, H. and Lu, J., 2011. Induction of apoptosis and inhibition of cell growth by tbx5 knockdown contribute to dysmorphogenesis in Zebrafish embryos. Journal of biomedical science, 18(1), pp.1-10.

McCurley, A.T. and Callard, G.V., 2008. Characterization of housekeeping genes in zebrafish: male-female differences and effects of tissue type, developmental stage and chemical treatment. BMC molecular biology, 9(1), pp.1-12.

Mukhopadhyay, D., & Chattopadhyay, A. (2014). Induction of oxidative stress and related transcriptional effects of sodium fluoride in female zebrafish liver. Bulletin of environmental contamination and toxicology, 93(1), 64-70.

Murtha JM, Keller ET. Characterization of the heat shock response in mature zebrafish (Danio rerio). Exp Gerontol. 2003 Jun;38(6):683-91. doi: 10.1016/s0531-5565(03)00067-6. PMID: 12814804.

Sarkar, S., Mukherjee, S., Chattopadhyay, A. and Bhattacharya, S., 2014. Low dose of arsenic trioxide triggers oxidative stress in zebrafish brain: expression of antioxidant genes. Ecotoxicology and environmental safety, 107, pp.1-8.

Stockhammer, O.W., Zakrzewska, A., Hegedûs, Z., Spaink, H.P. and Meijer, A.H., 2009. Transcriptome profiling and functional analyses of the zebrafish embryonic innate immune response to Salmonella infection. *The Journal of Immunology*, *182*(9), pp.5641-5653.

Uribe, R.A., Kwon, T., Marcotte, E.M. and Gross, J.M., 2012. Id2a functions to limit Notch pathway activity and thereby influence the transition from proliferation to differentiation of retinoblasts during zebrafish retinogenesis. Developmental biology, 371(2), pp.280-292.

Wang W-L, Hong J-R, Lin G-H, Liu W, Gong H-Y, Lu M-W, et al. (2011) Stage-Specific Expression of TNFα Regulates Bad/Bid-Mediated Apoptosis and RIP1/ROS-Mediated Secondary Necrosis in Birnavirus-Infected Fish Cells. PLoS ONE 6(2): e16740.

Wang, X.H., Souders 2nd, C.L., Zhao, Y.H., Martyniuk, C.J. 2018. Paraquat affects mitochondrial bioenergetics, dopamine system expression, and locomotor activity in zebrafish (Danio rerio). Chemosphere. 191, 106-117.

Watzke, J., Schirmer, K. and Scholz, S., 2007. Bacterial lipopolysaccharides induce genes involved in the innate immune response in embryos of the zebrafish (Danio rerio). *Fish & shellfish immunology*, *23*(4), pp.901-905.

Wu, S., Ji, G., Liu, J., Zhang, S., Gong, Y. and Shi, L., 2016. TBBPA induces developmental toxicity, oxidative stress, and apoptosis in embryos and zebrafish larvae (Danio rerio). Environmental toxicology, 31(10), pp.1241-1249.

Yang, L., Ivantsova, E., Souders II, C.L. and Martyniuk, C.J., 2021. The agrochemical S-metolachlor disrupts molecular mediators and morphology of the swim bladder: Implications for locomotor activity in zebrafish (Danio rerio). Ecotoxicology and Environmental Safety, 208, p.111641.

Zhang, H., Shen, B., Wu, H., Gao, L., Liu, Q., Wang, Q., Xiao, J. and Zhang, Y., 2014. Th17-like immune response in fish mucosal tissues after administration of live attenuated Vibrio anguillarum via different vaccination routes. *Fish & shellfish immunology*, *37*(2), pp.229-238.
